# Dorsal Dislocation of the Intermediate Cuneiform with a Medial Cuneiform Fracture: A Case Report and Review of the Literature

**DOI:** 10.1155/2013/238950

**Published:** 2013-09-25

**Authors:** Burak Akan, Tugrul Yildirim

**Affiliations:** ^1^Department of Orthopedics and Traumatology, Faculty of Medicine, Ufuk University, Ankara, Turkey; ^2^Dikmen C Parkpinar Evleri, 9/B No. 28 Keklikpinari Cankaya, 06450 Ankara, Turkey

## Abstract

Dorsal dislocation of the intermediate cuneiform and isolated medial cuneiform fractures are rare injuries. In this report, we present a patient who sustained a dislocation of the intermediate cuneiform and describe predisposing factors and the treatment procedure.

## 1. Introduction 

Dorsal dislocation of the intermediate cuneiform is a rare injury, and only a few cases have been reported [1–3]. The intermediate cuneiform is wedge shaped, lies between the medial and lateral cuneiforms, and is strongly attached to the first metatarsal. It is recessed at the second metatarsal base and forms the “keystone” of the Lisfranc tarsometatarsal joint complex [1]. Because it is wedge shaped and positioned dorsally, it has a tendency to dislocate dorsally, particularly when a plantar flexion force is applied to the midfoot [4]. Isolated cuneiform fractures are rarely observed and represent 1.7% of all tarsal fractures [5]. We present a case of a dorsally dislocated intermediate cuneiform and a fracture at the medial cuneiform. To our knowledge, dorsal dislocation of the intermediate cuneiform with a medial cuneiform fracture has not been previously reported in the literature. 

## 2. Case Report

**Figure 1:**
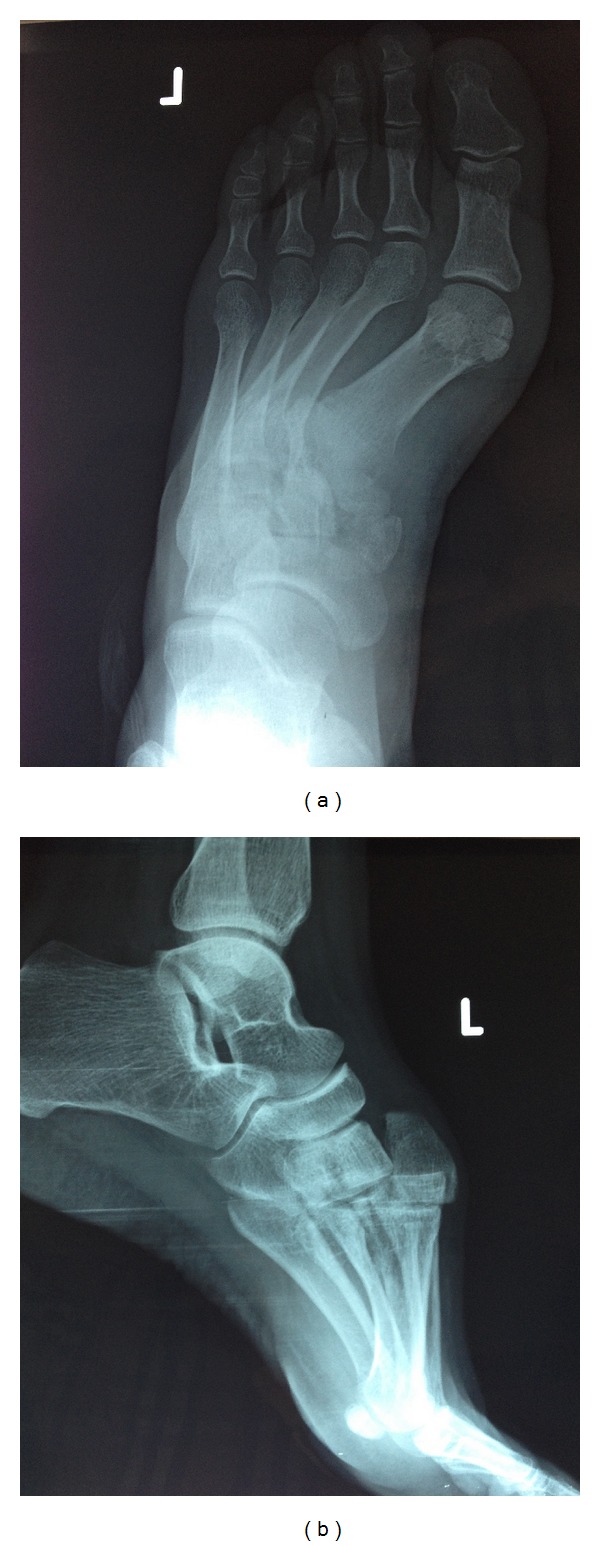
(a) Preoperative anteroposterior X-ray view. (b) Preoperative lateral X-ray view.

**Figure 2:**
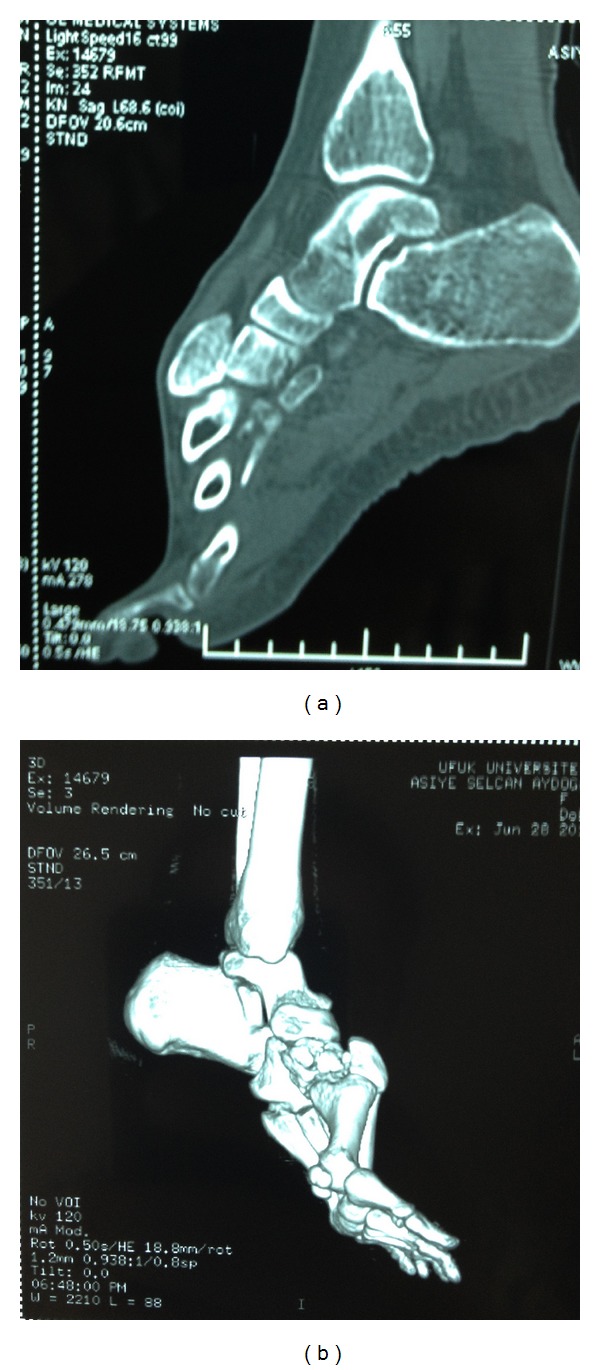
(a) Preoperative sagittal scan of computed tomography. (b) Preoperative three-dimensional reconstruction of computed tomography.

**Figure 3:**
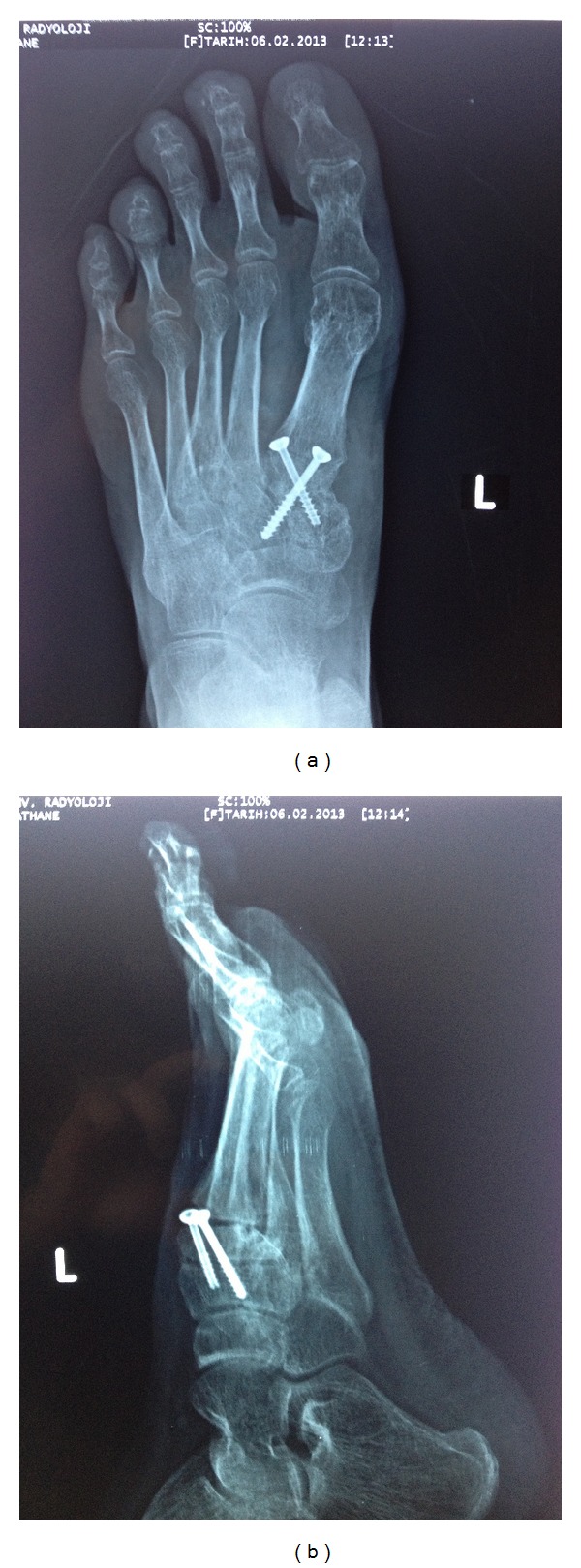
(a) Anteroposterior X-ray view 7 months after surgery. (b) Lateral X-ray view 7 months after surgery.

A 30-year-old woman sustained an injury to her right foot when she was walking in high-heeled shoes and fell down the stairs with her foot in an equinus and inversion position. The patient complained of severe pain and was unable to bear weight in her right foot. The initial clinical examination of her foot revealed swelling and tenderness at the dorsum of the midfoot without an open wound. There was no vascular compromise, and sensation was preserved. Plain radiographs showed dorsal dislocation of the intermediate cuneiform bone and a nondisplaced fracture at the medial cuneiform (Figure 1). A computed tomographic scan with three-dimensional reconstruction supported the radiographical findings (Figure 2). The patient was taken to the operation room, and a closed reduction was attempted under general anesthesia, but it did not succeed. The dorsal longitudinal approach was then performed. Open reduction was performed with fluoroscopic control, and two 3.5 mm cortical lag screws were used for stabilization. The postoperative radiographs were satisfactory (Figure 3). A short leg posterior splint in the neutral plantigrade position was applied for three weeks. The active range of ankle motion started 3 weeks after surgery. The patient was advised to avoid weight-bearing activities for 6 weeks. At 3 months after surgery, plain radiographs revealed fusion of the medial cuneiform and no recurrence of the intermediate cuneiform dislocation. At the final follow-up, the patient had painless foot with normal range of motion. Screws were removed 12 months after surgery at the patient's own request. 

## 3. Discussion 

The three cuneiforms are wedge shaped and sit in the middle of the medial column of the foot. They are part of the transverse and medial longitudinal arches of the foot. The intermediate cuneiform is the smallest. Each cuneiform articulates with one third of the distal navicula proximally and its respective metatarsal distally [6]. The stability of these bones is achieved by the deep transverse, dorsal, and plantar ligaments. The plantar ligaments are strengthened by the tibialis posterior tendon. The intercuneiform joints are planar-type joints that permit only gliding and rotation during pronation and supination movements [7, 8]. 

Isolated intermediate cuneiform dislocation was first described by Clark and Quint in 1933 [9]. In the literature, 13 cases of intermediate cuneiform dislocations have been associated with midfoot fractures: 10 were dorsal dislocations, and 3 were plantar [10]. Because the wedge shape is dorsally based, the intermediate cuneiform has a tendency to dislocate dorsally. Most intermediate cuneiform dislocations result from direct injury, but in some cases, indirect injury may cause the dislocation [11]. Nishi et al. performed an anatomic dissection to identify the mechanism of injury and the resultant pattern. According to their study, when the midfoot was under plantar flexion, the intermediate cuneiform was displaced dorsally, but when similar plantar flexion was applied under dorsal midfoot pressure, the intermediate cuneiform was prevented from dislocating dorsally [4]. In our opinion, dorsal shoe support helps maintain joint stability. Our patient was wearing high-heeled shoes with no dorsal support when she fell, and the intermediate cuneiform was therefore easily dislocated. Obtaining a detailed history of the mechanism of injury is important because the radiographic appearance of dislocation is confusing. The midtarsal foot anatomy is complicated with overlapping articulations that result in superimpositions on radiographical images [5]. In our case, we used computed tomography with three-dimensional reconstruction to determine the direction of dislocation and the displacement of the fracture to prevent overlooking another dislocation or fracture. 

In this case, closed reduction under general anesthesia failed, and we had to reduce openly. Five of the 6 reported cases with intermediate cuneiform dislocation were treated by open reduction [1, 2]. Although the failure rates of closed reduction are high, it should be attempted before open procedures. In conclusion, knowing the patient's choice of shoe is important for determining the mechanism of the injury. Immobilization and the avoidance of weight-bearing activity should continue for at least 6 weeks after surgery for successful treatment.
